# Editorial Change

**Published:** 1886-06

**Authors:** 


### Editorial Change.

We see by a recent circular and notice that Dr. Frank L. James, heretofore connected with the *National Druygist*, of St. Louis, has severed his connection with that journal, and is now a member of the editorial department of the St. Louis *Medical and Surgical Journal*. It will be remembered that Dr. James' special work on the *Druggist* was in the line of microscopy and therapeutics. The work in his new position will embrace the same subjects. This will enlist the attention of all who are specially interested in these branches in the *Medical and Surgical Journal*.

This is a medical journal that we can highly recommend to the dental profession, as it always contains matter of interest to medical men in whatever specialty they may be engaged.

Dentists are, perhaps, too much inclined to confine their professional reading to dental journals. We will venture here to suggest that while they should not read dental journals less, they should read medical journals more. It would be of advantage in many ways.

